# Investigation of Monte Carlo simulations of the electron transport in external magnetic fields using Fano cavity test

**DOI:** 10.1016/j.zemedi.2022.07.002

**Published:** 2022-08-25

**Authors:** Mohamad Alissa, Klemens Zink, Damian Czarnecki

**Affiliations:** a Institute of Medical Physics and Radiation Protection, University of Applied Sciences Giessen (THM), Giessen, Germany; b Department of Radiotherapy and Radiation Oncology, University Medical Center Giessen and Marburg, Marburg, Germany; c Marburg Ionbeam Therapycenter (MIT) Marburg, Germany

**Keywords:** Monte Carlo simulation, Fano cavity test, Detector, Dosimetry in external magnetic fields

## Abstract

**Purpose:**

Monte Carlo simulations are crucial for calculating magnetic field correction factors $k_{B}$ for the dosimetry in external magnetic fields. As in Monte Carlo codes the charged particle transport is performed in straight condensed history (CH) steps, the curved trajectories of these particles in the presence of external magnetic fields can only be approximated. In this study, the charged particle transport in presence of a strong magnetic field $\overset{\to }{B}$ was investigated using the Fano cavity test. The test was performed in an ionization chamber and a diode detector, showing how the step size restrictions must be adjusted to perform a consistent charged particle transport within all geometrical regions.

**Methods:**

Monte Carlo simulations of the charged particle transport in a magnetic field of 1.5 T were performed using the EGSnrc code system including an additional EMF-macro for the transport of charged particle in electro-magnetic fields. Detailed models of an ionization chamber and a diode detector were placed in a water phantom and irradiated with a so called Fano source, which is a monoenergetic, isotropic electron source, where the number of emitted particles is proportional to the local density.

**Results:**

The results of the Fano cavity test strongly depend on the energy of charged particles and the density within the given geometry. By adjusting the maximal length of the charged particle steps, it was possible to calculate the deposited dose in the investigated regions with high accuracy (<0.1%). The Fano cavity test was performed in all regions of the detailed detector models. Using the default value for the step size in the external magnetic field, the maximal deviation between Monte Carlo based and analytical dose value in the sensitive volume of the ion chamber and diode detector was 8% and 0.1%, respectively.

**Conclusions:**

The Fano cavity test is a crucial validation method for the modeled detectors and the transport algorithms when performing Monte Carlo simulations in a strong external magnetic field. Special care should be given, when calculating dose in volumes of low density. This study has shown that the Fano cavity test is a useful method to adapt particle transport parameters for a given simulation geometry.

## 1 Introduction

Integrating magnetic resonance tomography (MRI) with medical linear accelerators allows monitoring the tumour during radiotherapy treatment [1], [2], [3], [4], [5]. Due to the Lorentz force, the magnetic field impacts the trajectories of the secondary charged particles, affecting both the dose distribution and the dose response of a detector. Current Monte Carlo methods accurately describe the radiation transport in different materials, even in the presence of a magnetic field. Therefore, they are the ideal approach for evaluating the impact of magnetic fields on clinical dosimetry [6], [7], [8]. However, Monte Carlo codes use condensed history steps to calculate the trajectory of charged particles [9]. Therefore, trajectories of charged particles determined by Monte Carlo simulations are an approximation of the real particle trajectory. Considering the way currently available Monte Carlo algorithms account for charged particle transport in external magnetic fields, approximations are made that may affect the electron path. When treating the charge particle scattering and magnetic field deflection as independent processes, the step size of the charge particle must be restricted. Otherwise, there is a possibility that a bias may occur in the particle transport [10], especially if several interactions are combined in a single particle transport step (condensed history step).

Today, many general purpose Monte Carlo codes like GEANT4, PENELOPE, MCNP6 or EGSnrc are able to describe the charged particle transport in external electric or magnetic fields [11]. For the EGSnrc code system two different macros for this purpose exist: a version called *emf*_*macros.mortran* (‘EMF’), available in EGSnrc already since the transition from EGS4 to EGSnrc. This macro is based on the theory proposed by Bielajew [12]. A more sophisticated macro called *eemf*_*macros.mortran* (‘EEMF’) was introduced in 2017 by Malkov and Rogers [13]. Within these macros the single scattering mode used in the vicinity of interfaces was improved for the charged particle transport in presence of a Lorentz force. Moreover, an improved boundary crossing algorithm (BCA) was implemented. Both improvements were implemented to avoid artifacts when particles cross boundaries [14]. When particles approach an interface, Monte Carlo transport algorithms typically switch from multi scatter to single scatter mode. The B-field does not change the nearest distance to the next boundary of a region, but the trajectory can be bent to such an extent that a region may be skipped. This can occur especially with very complex geometries and regions of very low density and can result in incorrect dose calculations in individual regions of the geometry. The Fano theorem [15] plays an important role in Monte Carlo simulations of the response of gas-filled ion chambers by providing a consistency test of the particle transport. This test is the only known method allowing the validation of charged particle energy deposition in heterogeneous media against an analytic expression, this way testing the charged particle step algorithm in the given geometry and also the bounding crossing algorithm [16]. According to the recommendations of the AAPM TG-268 report [17] a Fano test is strongly recommended when reporting Monte Carlo calculated results of detectors with gaseous cavities. In the presence of external magnetic fields where the trajectories of the charged particles are more complex due to the Lorentz force, Fano’s theorem may also be applied, but its validity prerequisites special conditions for the primary particle source, the isotropy and spatial uniformity of the source [14], [16].

There are several studies investigating the consistency of Monte Carlo transport algorithms in the presence of a magnetic field using the Fano cavity test. Pooter *et al.* [14] used a simplified geometry of a Farmer-type ionization chamber consisting only of an air-filled cavity and a surrounding wall. The results suggest that a comparable accuracy to Monte Carlo simulations without a B-field may not be achieved in presence of a B-field with the investigated Monte Carlo algorithms. The authors recommend that each simulation geometry and set-up should be carefully validated before use. Lee *et al.* [11] compared the charged particle transport of different Monte Carlo algorithms (EGSnrc, Geant4, PENELOPE and MCNP6). They studied the electron transport in the energy range from 0.01 MeV to 3 MeV in different magnetic field strengths from 0 T to 3 T, showing that care should be taken when the step size of the electron transport is in the range of the Larmor radius $r_{G}$ of the electrons. They also investigated the dose deposition in a cylindrical gas-filled disk between two solid walls. Ito *et al.* [18] compared the EGS5 and the EGSnrc codes with the above mentioned EEMF macros. They evaluated the accuracy of the charged particle transport in external B-fields of 0.35 and 1.5 T within a simple cylinder geometry. Electrons with energies between 0.01 and 10 MeV were used for the Fano source.

While Lee *et al.* [11] used the egs_chamber code in his study, Malkov *et al.* used the DOSRZnrc user code for simple geometries like a gas slab of 0.2 and 2 cm thicknesses. In both studies an accuracy of 0.1% in the Fano test was achieved. Ito *et al.* used the Fano cavity test with EEMF macros to evaluate the accuracy of electron transport in 0.35 and 1.5 T for EGS5 code. They simulated a simple cylinder made of three layers, the energy of the Fano source was varied between 0.01 to 10 MeV [18]. In further Monte Carlo based studies calculating the detector response in the presence of external magnetic fields [4], [19], [20], [21] the authors performed the Fano test only with one electron energy and investigated only the sensitive volume of the detector.

So, most of the existing studies using the Fano theorem to investigate Monte Carlo radiation transport in external magnetic fields were limited to highly simplified ionization chamber geometries. It remains an open question whether Monte Carlo algorithms can achieve comparable accuracy’s for a Fano test for complex detector geometries. A Fano test of all regions of a detector model and not only for the sensitive volume of the detector, might be necessary under the following hypothetical circumstances: Suppose there exists a region A outside the sensitive volume in which there is a statistically significant deviation from the expected value under Fano conditions, but this has no effect on the sensitive volume. This leads to the conclusion that on the one hand the radiation transport in and out of this region is calculated incorrect, but on the other hand this has hardly any influence on the dose contribution in the sensitive volume when using a radiation source which fulfills the Fano conditions. But if the detector model is positioned in a clinical radiation field in such a way that region A is the largest source of secondary electrons scattering into the sensitive volume, this may lead to an incorrectly calculated dose in the sensitive volume, even though in the Fano cavity test the dose in the sensitive volume was in agreement with the expected values.

The objective of this work was to investigate the particle transport in presence of a strong magnetic field using the Fano test. The consistency of charged particle transport in dependence of the maximum step size of the charged particles was investigated in all geometrical regions of two detailed detector models, an ionization chamber and a Si diode, with the question, which geometrical regions of a detector model are most critical with regard to particle transport in external magnetic fields. Since the radius of the curved trajectory of the charged particles depends on their energy, the Fano test was performed for different primary electron energies.

## 2 Materials and methods

### 2.1 Theoretical background

#### 2.1.1 Basic description of electron trajectories in external magnetic fields

Considering an electron moving in the direction $\overset{\to }{u}$ in an external magnetic field $\overset{\to }{B}$ in vacuum, the change of particle direction d$\overset{\to }{u}$ with the path length d*s* of the particle can be described as follows,

(1) $$\frac{d\overset{\to }{u}}{ds}=\frac{e}{m_{0}\;c\;\beta \;\gamma }\overset{\to }{u}\times \overset{\to }{B}$$

where *e* is the elementary charge, $m_{0}$ the electron rest mass, β the relative electron velocity with respect to the speed of light *c* and γ is the Lorentz factor [22]. From Eq. (1) it can be seen that the influence of the magnetic field on the particle trajectory is energy dependent. To estimate the order of magnitude of the particle deflection, the Larmor radius or gyroradius $r_{G}$ of the electron can be calculated from Eq. (1) as follows:

(2) $$r_{G}=\frac{m_{0}\;c\;\beta \;\gamma }{e\left|\overset{\to }{u}\times \overset{\to }{B}\right|}$$

**Figure 1:**
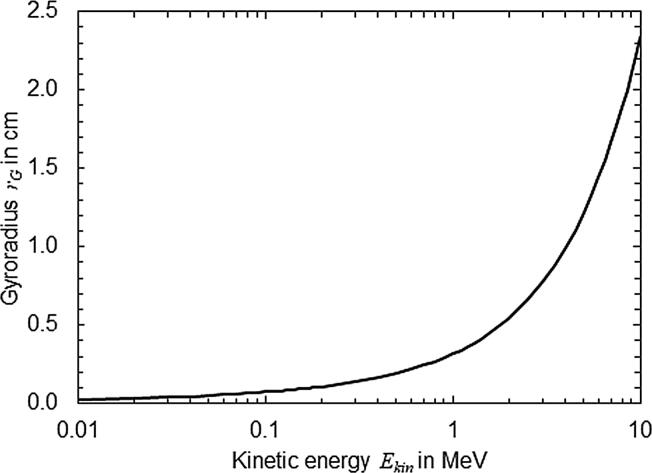
The gyroradius $r_{G}$ of an electron moving perpendicular to an external magnetic field (*B* = 1.5 T) as a function of $E_{\mathrm{kin}}$.

The radius $r_{G}$ is shown in Fig. 1 as a function of the kinetic energy of an electron in a magnetic field perpendicular to the direction of movement with a magnetic field strength of 1.5 T. As can be seen, the gyroradius $r_{G}$ for low energy electrons (< 1 MeV) is within the order of magnitude of the components of an ionization chamber (sensitive volume, central electrode etc.). This means that special care should be taken when the low-energy electron transport is simulated through an ionization chamber. The circular path of kV-electrons is in the range of a few millimeters. For high-energy electrons, on the other hand, the influence of the magnetic field is smaller.

Class II Monte Carlo algorithms simulate charged particle transport in condensed history (CH) steps, summarizing multiple elastic scattering events in one single transport step. A CH step-length depends on the density ρ of the medium in which particles are transported, since the probability of hard collisions increases with ρ. A more detailed description of the particle transport algorithm can be found in the work of Berger [23].

With respect to particle transport in CH steps in external magnetic fields, it is useful to look at the directional change d*u* with the mass-path length ρd*s* to account for the density dependence of the interaction probability along the path length. This transforms Eq. (1) as follows

(3) $$\frac{d\overset{\to }{u}}{\rho \;ds}=\frac{e}{\rho \;m_{0}\;c\;\beta \;\gamma }\overset{\to }{u}\times \overset{\to }{B}$$

According to this equation, it can directly be seen that the curvature of an electron trajectory depends on the density of the medium, when the trajectory is observed in mass-thickness spatial coordinates $\rho \;\overset{\to }{x}$. This was very well elaborated in the work of Bouchard *et al.* [16]. Thus, there is a reciprocal dependence of the change of direction d$\overset{\to }{u}$ on the density of the medium ρ. From Eq. 3 it is clear that the influence of the external magnetic field on the particle trajectory increases in media with decreasing density and with decreasing particle energy. Thus, special care should be given when the radiation transport of low-energy charged particles is calculated by class II Monte Carlo simulation algorithms in regions of low density ρ in an external magnetic field $\overset{\to }{B}$.

#### 2.1.2 Condensed history steps in external magnetic fields

Regarding the charged particle transport using CH steps in external magnetic fields, the following approximations have to be respected. In the work of Bielajew *et al.* [10], a general expression for a CH step is formulated, from which the velocity $\overset{\to }{v}$ of a charged particle can be calculated after a transport step *s* in a homogeneous medium:

(4) $$\overset{\to }{v}={\overset{\to }{v}}_{0}+\frac{1}{m_{0}\;\gamma }\int_{0}^{t}{\overset{\to }{F}}_{\mathrm{ret}}(E(t))+{\overset{\to }{F}}_{\mathrm{ms}}(E(t))+{\overset{\to }{F}}_{L}(\overset{\to }{x}(t^{\prime }),E(t),\overset{\to }{u}(t^{\prime }))dt^{\prime }$$

where ${\overset{\to }{v}}_{0}$ is the velocity of the particle before the step *s* and *t* is the time interval of the step. ${\overset{\to }{F}}_{\mathrm{ret}}$ and ${\overset{\to }{F}}_{\mathrm{ms}}$ are the forces from inelastic and multiple scattering, respectively. Here, the Lorenz force of the external magnetic field $\overset{\to }{B}$ is referred to as ${\overset{\to }{F}}_{L}$. All forces ${\overset{\to }{F}}_{\mathrm{ret}},{\overset{\to }{F}}_{\mathrm{ms}}$ and ${\overset{\to }{F}}_{L}$ acting on the electron, have an particle energy *E* dependency. For uniform magnetic fields, the dependence of the Lorenz force ${\overset{\to }{F}}_{L}$ on the location $\overset{\to }{x}$ of the particle does not exist. An important aspect of Monte Carlo simulations in CH steps is to keep the steps *s* small enough so that the energy dependence *E* of the forces acting on the particle is negligible. For particle transport in a homogeneous magnetic field, the change in particle direction $\overset{\to }{u}$ must also be as small as possible, so that the equation can be simplified as follows:

(5) $$\overset{\to }{v}={\overset{\to }{v}}_{0}+\frac{1}{m_{0}\;\gamma }\left({\overset{\to }{F}}_{\mathrm{ret}}(E_{0})+{\overset{\to }{F}}_{\mathrm{ms}}(E_{0})+{\overset{\to }{F}}_{L}(E_{0},{\overset{\to }{u}}_{0})\right)$$

with $E_{0}$ the initial energy and ${\overset{\to }{u}}_{0}$ the propagation direction of the particle. To ensure that the assumptions leading to Eq. (5) do not lead to transport artefacts, the length of the path *s* must be adjusted or checked with respect to the magnitude of d$\overset{\to }{u}$. An inaccurate trajectory of a particle would not necessarily lead to a miscalculated dose deposition if the particle would never leave its geometric region due to energetic reasons. Problems occur when particles cross borders of different regions. Critical parameters with respect to the length of a CH step are the energy (or velocity) of the charged particle and the density of the medium in which the particle transport is calculated [22].

#### 2.1.3 Fano cavity test

If the particle trajectory is only approximated, it is not clear whether in between a single CH step the particle might have interacted in another region of a different medium. For this reason, a self-consistency test of the charged particle transport algorithm based on the Fano theorem was developed for Monte Carlo calculations [24]. The Fano test states that under charged particle equilibrium and for uniform cross-sections, the fluence of the charged particles is independent of the mass density [15]. If we consider charged particles in an external magnetic field, the Fano conditions are violated because, unlike the other forces in Eq. (5), the Lorenz force does not scale with mass density [25].

For this reason, Bouchard *et al.* [16] have proposed further special conditions under which the Fano theorem remains valid in the presence of an external magnetic field. Either the radiation source must be spatially uniform and isotropic so that the Fano conditions are satisfied for any external magnetic field, or the intensity of the magnetic field must be scaled with density. In this work we used a spacial uniform and isotropic radiation source to perform the Fano cavity test in an external uniform magnetic field, The Fano radiation source used in this work generated electrons propagating uniformly in all directions within a given rectangular volume. The size of the volume was chosen according to the energy of the electrons: (7×5×5) cm^3^ for 0.1 and 1 MeV and (10×9×9) cm^3^ for 6 MeV electrons. The detector was placed at the center of this volume.

### 2.2 Monte Carlo simulation set-up

This study is based on Monte Carlo simulations performed using the EGSnrc code system [26] including the standard EGSnrc macro *emf*_*macros.mortran* [12] (‘EMF’) for transporting charged particles in external magnetic and electric fields. The electron transport was investigated for different EM ESTEPE values from 0.25 to as low as 0.005. The EM ESTEPE value within the EMF macros is used to control the maximum step length *s* according to the equation:

(6) $$s=(\mathrm{EM}\;\mathrm{ESTEPE})\times r_{G}$$

i.e. the step length in presence of an external magnetic field is limited to a fraction of the Larmor radius $r_{G}$. This ensures, that the step size *s* is always adapted to the *B*-field and the particle energy. For Monte Carlo simulations without an external $\overset{\to }{B}$-field, the parameter EM ESTEPE has no relevance, the normal step-size parameter ESTEPE was set to the default value ESTEPE = 0.25, meaning that the maximum energy loss within one CH step is 25%. Further details of the investigated Monte Carlo simulation set-up are summarized in Table 1.

**Table 1:** Summary of the main properties and parameters for the Monte Carlo simulations with EGSnrc in this work.

Item	Description	References
Code	EGSnrc 2020 master brunch	Kawrakow *et al.*[26]
	egs++ library	Kawrakow *et al.*[27]
	egs_chamber	Wulff *et al.*[28]
	emf_macros.mortran	Bielajew *et al.*[10]
Timing	See Table 3	
Source description	egs++ egs_fano_source surrounding the detector to achieve charged particle equilibrium within the detector geometry; initial particle energies: monoenergetic electrons with energies 0.1 MeV, 1 MeV and 6 MeV	Kawrakow *et al.*[27]
Cross-sections	XCOM photon cross section with multiconfiguration DiracFock renormalization factor for the photoelectric effect (mcdf-xcom)	
Transport parameters	Boundary crossing algorithm: Exact, transport and particle production threshold energy of 512 keV and 1 keV for electron and photon, respectively; EM ESTEPE = 0.25–0.005	
Variance reduction techniques	Russian Roulette range rejection technique with a survival probability of 1/128	
Statistical method	History-by-history	
Post-processing	None	

The EM ESTEPE value also impacts the efficiency ∊ of a Monte Carlo simulation, where ∊ is given as:

(7) $$∊=\frac{1}{T\;\sigma^{2}}$$

*T* is the CPU time and σ the type-A relative standard uncertainty of the Monte Carlo calculated quantity.

### 2.3 Detector models

**Figure 2:**
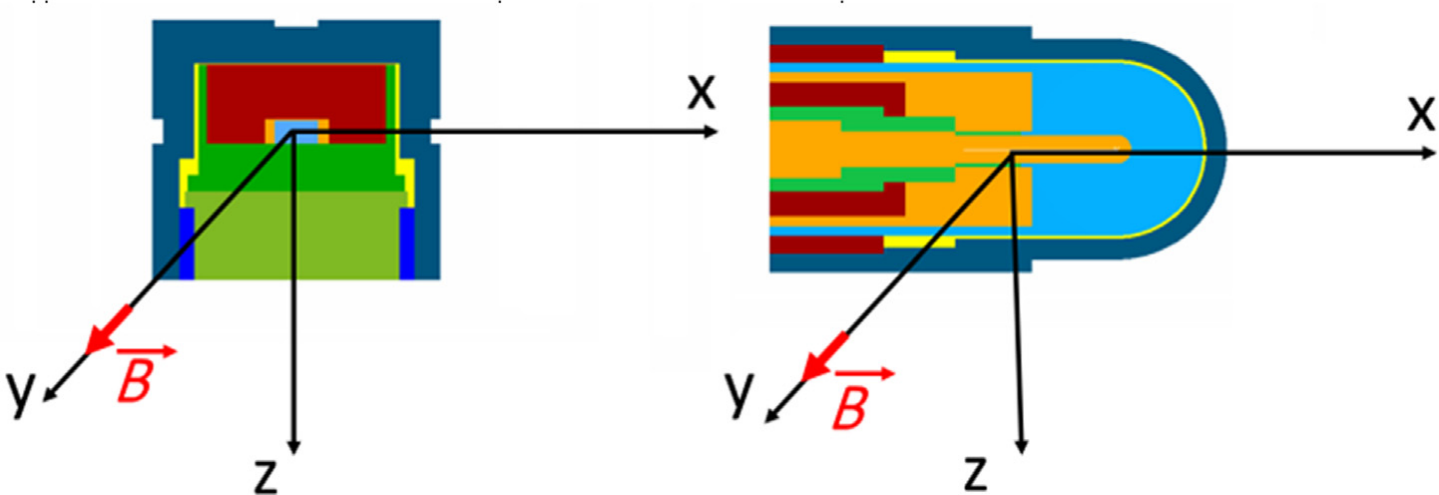
Cross sections of the Monte Carlo based model of the investigated ionization chamber PTW 31021 and the Si diode T60016. Different colours represent different materials. The red arrow represents the orientation of the external magnetic field $\overset{\to }{B}$.

In this study, the SemiFlex 3D ionization chamber (PTW 31021) and the diode detector (PTW T60016) from PTW (Freiburg, Germany) have been investigated. They were modelled in detail according to manufacturer data using the egs++ class library [27]. Cross-sections of the detector models are shown in Fig. 2. The ionization chamber has a sensitive air volume of 0.07 cm^3^. The electrode is made of aluminium with a radius 0.04 cm. The Monte Carlo model of the ionization chamber consists of 47 regions. The diode detector has a sensitive volume of silicon with a volume of 3.4×10^−4^${\mathrm{cm}}^{3}$. The Monte Carlo model of the diode detector consists of 30 regions. Table 2 presents the detector regions with the corresponding region numbers of the most important detector components.

**Table 2:** Detector components and corresponding region numbers and materials.

Detector	Detector component	Materials	Region number
PTW 31021	Sensitive volume	Air	2 and 6
	Central electrode	Aluminium	1 and 5
	Wall	Graphite, PMMA	3, 4, 7 and 8
	Stem	Various materials	9 to 47
T60016	Sensitive volume	Silicon	1

### 2.4 Configuration for Fano cavity test

All Monte Carlo simulations were performed under Fano conditions to test the consistency of charged particle transport in presence of an external magnetic field. To realize Fano conditions with an external magnetic field, the particles source egs_fano_source from the EGSnrc C++ class library has been used. This radiation source emits particles proportional to the mass density at the current source position with uniformly distributed direction in 4 π [27]. The detectors were placed in a water phantom large enough to enable charged particle equilibrium in the modeled detectors. With respect to the range of electrons, a phantom of size (12×10×10) cm^3^ was chosen for electrons with initial energies of 0.1 MeV and 1 MeV. For the 6 MeV electrons a larger phantom sized (20×20×20) cm^3^ had to be chosen. In addition, all materials of the investigated detector geometries were replaced by water with density of the original material. The density correction and I value were set to those of water for all materials so that the mass stopping power of all materials were identical. All calculations were performed in an external magnetic field of 1.5 T which was perpendicular to the symmetry axis of the detectors (see Fig. 2). With this simulation setup, the Fano conditions as described by Bouchard [16] could be satisfied even in the presence of the applied external magnetic field. Under these conditions, the Monte Carlo calculated absorbed Dose $D_{\mathrm{MC},i}$ in a region *i* is independent of the magnetic field strength and can be calculated according to the following equation:

(8) $$D_{\mathrm{MC},i}=n_{i}\frac{E_{0}}{m_{i}}$$

where $m_{i}$ is the mass of region *i* and $n_{i}$ is the number of particles emitted from the Fano source in region *i*. $E_{0}$ is the initial particle energy.

## 3 Results

### 3.1 Charged particle transport without external magnetic fields

**Figure 3:**
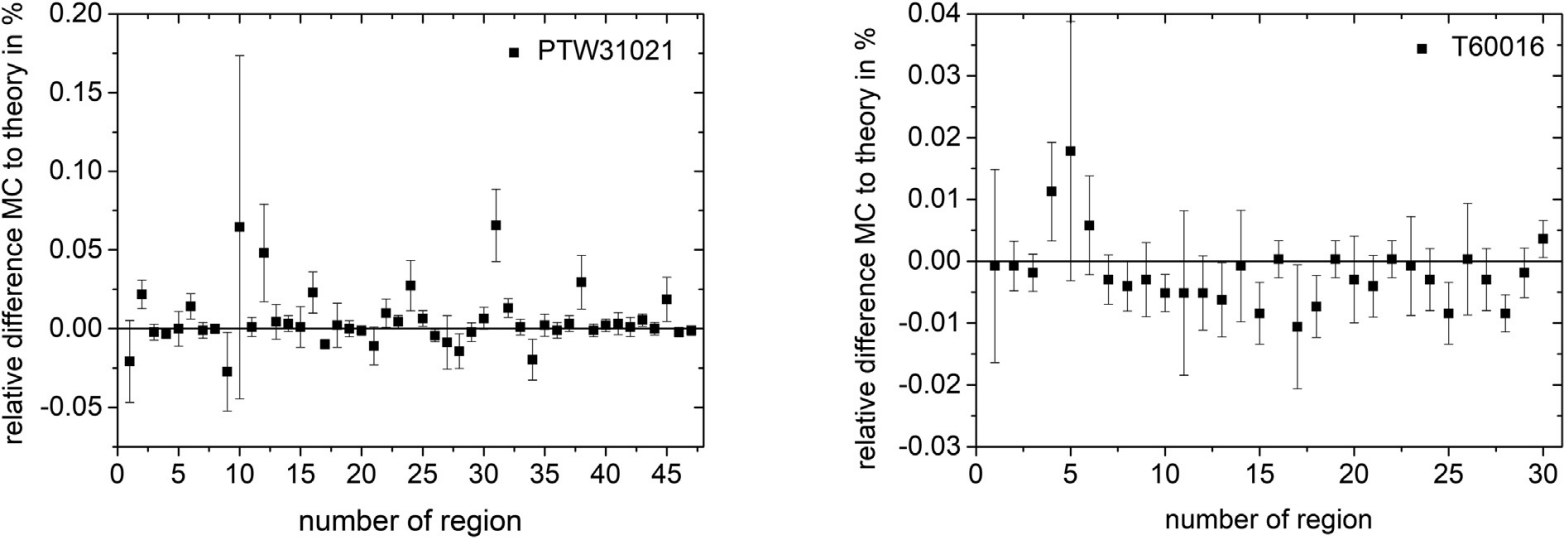
Relative difference of absorbed dose in all geometrical regions of the investigated ionization chamber PTW 31021 (a) and Si diode detector PTW 60016 (b) from the theoretical value under Fano conditions. Monoenergetic 1 MeV electrons were chosen as radiation source. The Monte Carlo simulations were performed with ESTEPE = 0.25 and without an external magnetic field. The type-A relative standard uncertainty of the Monte Carlo data is represented by uncertainty bars.

Fig. 3 shows the relative difference between the Monte Carlo calculated and expected dose in each region of the ionization chamber and the diode detector without an external magnetic field under Fano conditions. The transport parameter ESTEPE is set to the default value of 0.25. The Monte Carlo calculated absorbed dose in all regions of both detailed detector models show small deviations from theoretical values within 0.06%. The presented Fano test was performed using 1 MeV monoenergetic electrons as particle source.

### 3.2 Charged particle transport in external magnetic fields

#### 3.2.1 Diode detector

**Figure 4:**
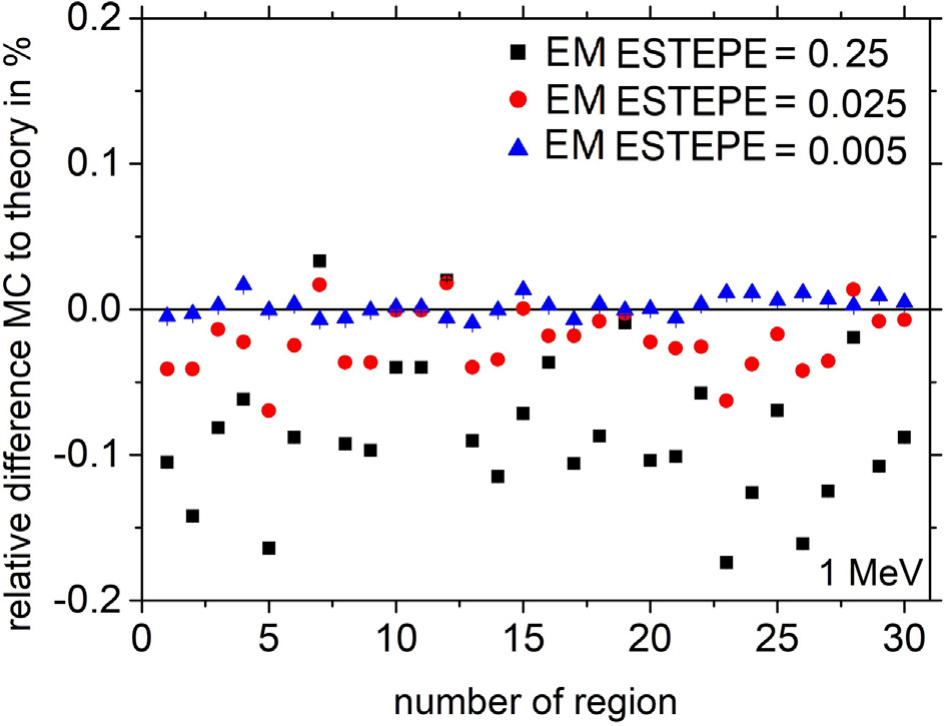
Relative difference of absorbed dose in all regions of the Si diode PTW 60016 from the theoretical value under Fano conditions. Monoenergetic 1 MeV electrons were chosen as radiation source. The Monte Carlo simulations were performed with different EM ESTEPE values in presence of an external magnetic field *B* = 1.5 T perpendicular to the symmetry axis of the detector. The type-A relative standard uncertainty of the Monte Carlo data is within the symbol size.

When the charged particle transport is calculated in the presence of an external magnetic field (B  = 1.5 T), the deviation from the theoretical dose value increases up to 0.11% within the active volume (region 1) for the Si diode detector (see Fig. 4) when a value for EM ESTEPE = 0.25 was used. Other regions of the diode detector showed comparable or even larger deviations from the theoretical value. The deviations could be significantly reduced when the EM ESTEPE value was decreased to 0.025 or 0.005. For both values the deviations between the theoretical and the Monte Carlo based dose value were well below 0.1% for all regions.

**Figure 5:**
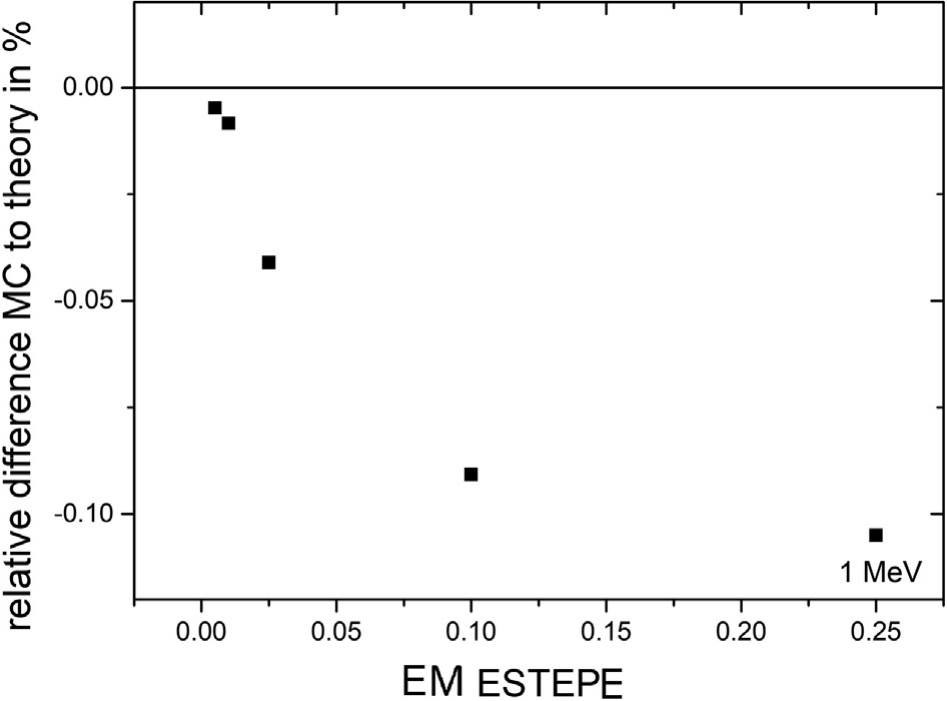
Relative difference of absorbed dose in the sensitve volume of the Si diode detector PTW 60016 from the theoretical value under Fano conditions as a function of the radiation transport parameter EM ESTEPE. Monoenergetic 1 MeV electrons were chosen as radiation source in presence of an external magnetic field *B* = 1.5 T perpendicular to the symmetric axis of the detector. The type-A relative standard uncertainty of the Monte Carlo data is within the symbol size.

Fig. 5 shows the relative dose deviation in the sensitive volume of the diode as a function of the EM ESTEPE value for electrons with an initial energy of 1 MeV. As can be seen, the deviations are within 0.1%. However, there is a clear relation between the dose values in the sensitive volume and the applied EM ESTEPE values.

#### 3.2.2 Ionization chamber

**Figure 6:**
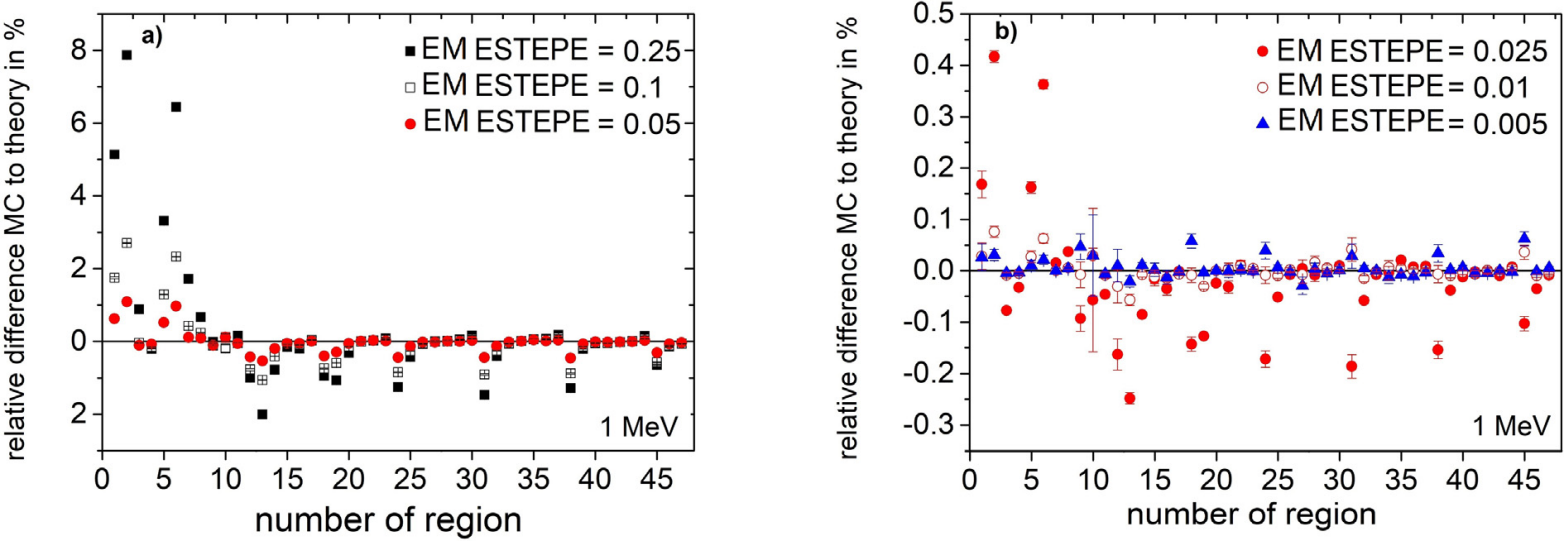
Relative difference of absorbed dose in all geometrical regions of the ionization chamber PTW 31021 from the theoretical value under Fano conditions. Monoenergetic 1 MeV electrons were chosen as radiation source. The Monte Carlo simulations were performed in presence of an external magnetic field *B* = 1.5 T oriented perpendicular to the symmetry axis of the detector. The type-A relative standard uncertainties of the Monte Carlo data are represented by uncertainty bars or they are given by the symbol size.

**Figure 7:**
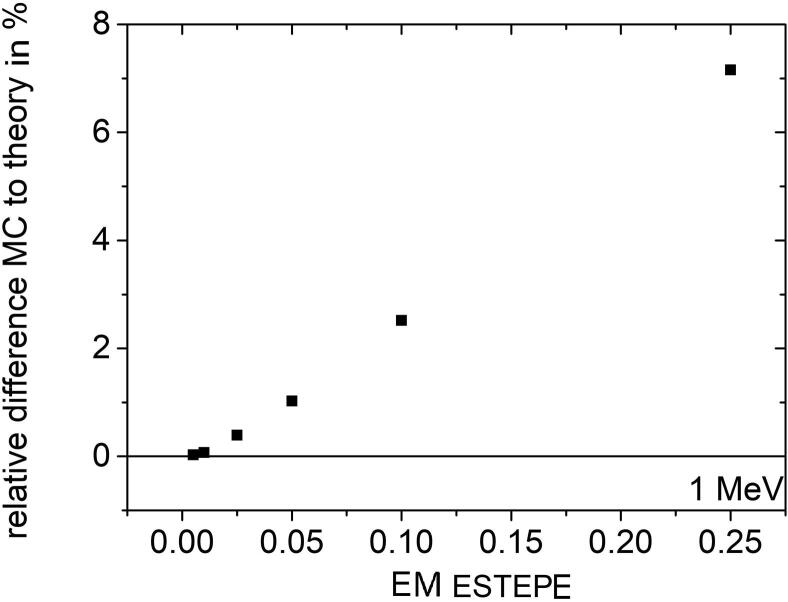
Mean value of the relative difference of absorbed dose from the theoretical value under Fano conditions as a function of EM ESTEPE for the two air-filled regions 2 and 6 of the ion chamber. Monoenergetic 1 MeV electrons were chosen as radiation source. The Monte Carlo simulations were performed with varying EM ESTEPE values in presence of an external magnetic field *B* = 1.5 T oriented perpendicular to the symmetry axis of the detector. The type-A standard uncertainties of the Monte Carlo data are given by the symbol size.

Fig. 6 shows the relative dose deviation between the expected and Monte Carlo calculated values in all geometrical regions of the ionization chamber PTW 31021 for various EM ESTEPE values. All results presented in Fig. 6 were calculated with 1 MeV monoenergetic electrons as particle source in an external magnetic field *B* = 1.5 T perpendicular to the symmetry axis of the ionization chamber (see Fig. 2). The largest deviation of the Monte Carlo based dose from the expected value is observed in the air-filled regions (2, 6, 10, 12, 18, 24, 31, 38 and 45) of the ion chamber. Among these regions, part of the sensitive volume of the ionization chamber (regions 6) showed the largest deviation of about 8%. It is the largest air-filled volume. Smaller air volumes in the chamber stem also show deviations of more than 1%. To achieve a deviation below 0.1% in all regions, the EM ESTEPE value had to be reduced to 0.01. By reducing the EM ESTEPE value to 0.005, the deviations could be further reduced. Regarding only the active, air-filled volume of the ion chamber (region 2 and 6) the importance of an appropriate EM ESTEPE factor especially in low density materials become clear (see Fig. 7). Decreasing EM ESTEPE from 0.25 to 0.01, the difference between Monte Carlo based and analytical dose value decreased from around 7% to 0.1% and below, i.e. the CH steps in low density materials has to be very small to have an adequate approximation of the curved trajectories of the electrons. The comparison of Figure 5, Figure 7 shows, that the relationship of the relative difference of Monte Carlo based and analytical dose value as a function of the EM ESTEPE value is different. Whereas for the Si-diode the difference is always negative, i.e. the Monte Carlo based dose value is smaller than the expected one, it is positive for the ion chamber. This behavior is not clear at the moment, but it is assumed that the difference is related to the different densities of the active volumes of the two detectors (air and silicon).

### 3.3 Fano test for various initial electron energies

**Figure 8:**
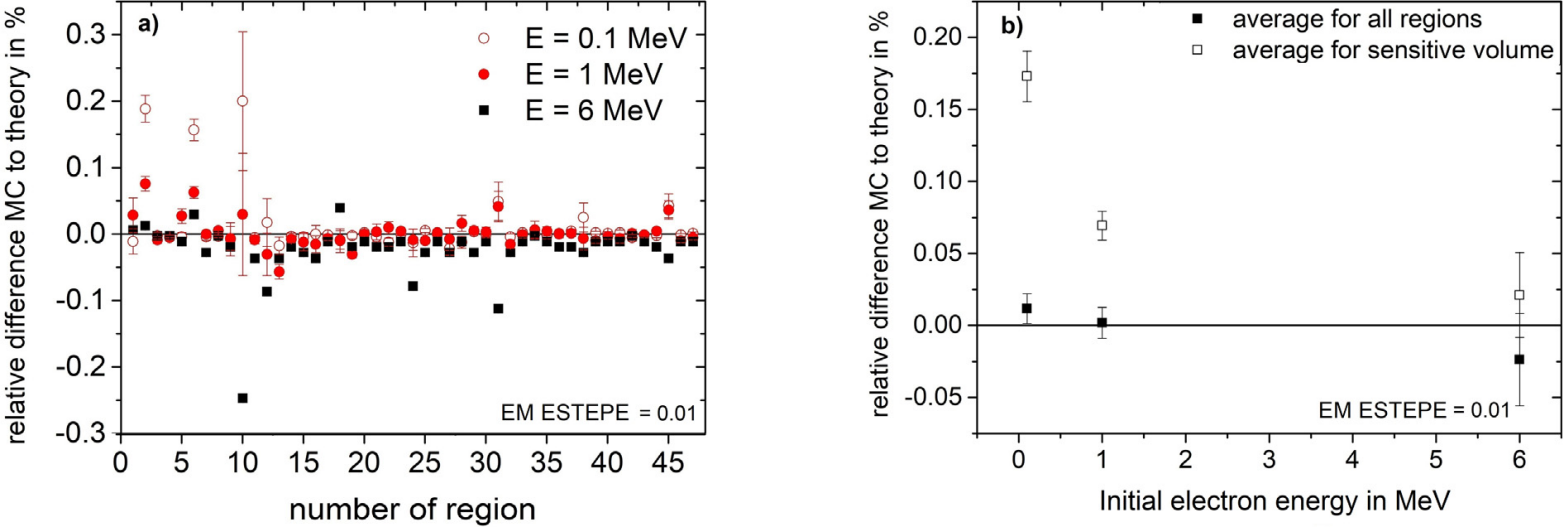
Relative difference of absorbed dose in all geometrical regions of the ionization chamber PTW 31021 from the theoretical value under Fano conditions in presence of an external magnetic field *B* = 1.5 T. In figure a) the EM ESTEPE value was set to 0.01 and the Fano test was performed with three different initial electron energies for the particle source. In figure b) the relative difference for the sensitive volume and the average of all regions is shown as a function of the initial electron energy of the radiation source for EM ESTEPE = 0.01. The type-A standard uncertainties of the data presented in panel a) and b) are given by uncertainty bars or are within the symbol size. The type-A standard uncertainties in panel b) are the combined uncertainties over the given regions.

**Figure 9:**
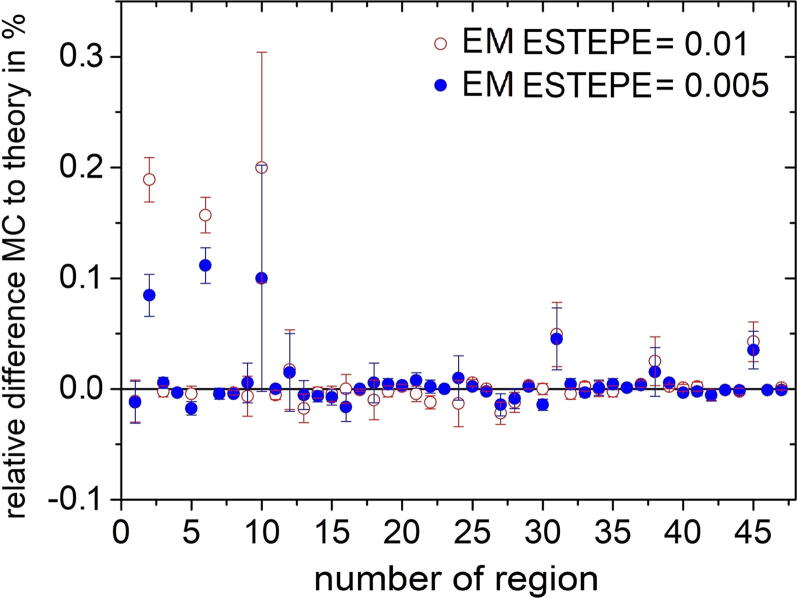
Relative difference of absorbed dose for all geometrical regions of the ionization chamber PTW 31021 from the theoretical value under Fano conditions for electrons with an initial electron energy of 0.1 MeV in presence of an external magnetic field *B* = 1.5 T. The relative dose difference is calculated for two different EM ESTEPE values. The type-A relative standard uncertainties of the Monte Carlo data are represented by uncertainty bars, or are within the symbol size.

Fig. 8 shows the results of the Fano cavity test for all geometrical regions of the ionization chamber for three different initial electron energies 0.1 MeV, 1 MeV and 6 MeV. As can be seen in Fig. 8 b, the deviation between the Monte Carlo calculated dose and the theoretical dose in the sensitive volume is smaller for electrons with an initial energy of 6 MeV and increases for electrons with lower initial energy. If the mean dose deviation over the two regions of the sensitive volume of the ion chamber is considered, one can see that even for the very small EM ESTEPE value of 0.01 the deviations are above 0.1% for the smallest electron energy of 0.1 MeV, i.e. in that case the Fano test has failed. Fig. 9 shows that the EM ESTEPE parameter had to be reduced to 0.005 to reduce the deviation between the Monte Carlo calculated and theoretical dose values in all regions of the ionization chamber below 0.1% for electrons with an initial energy of 0.1 MeV.

### 3.4 Efficiency of Monte Carlo simulations

Table 3 presents the calculation time *T*, the uncertainty σ and the efficiency ∊ according to Eq. 7 in dependency of the EM ESTEPE value for charged particle transport through the investigated detectors with and without an external magnetic field under Fano test conditions with monoenergetic electrons as radiation source. The magnetic field is oriented perpendicular to the symmetry axis of both detectors (see Fig. 2).

**Table 3:** Simulation efficiency for different parameters EM ESTEPE. The simulations were performed with an primary electron energy of 1 MeV for the Fano source.

	PTW 31021				PTW T60016			
*B*	EM ESTEPE	*T* in h	σ in %	∊	EM ESTEPE	*T* in h	σ in %	∊
1.5 T	0.25	2565	0.011	3.22	0.25	3320	0.004	18.83
	0.1	3169	0.011	2.61	0.1	3397	0.004	18.40
	0.05	3195	0.011	2.59				
	0.025	3291	0.011	2.51	0.025	3677	0.004	17.00
	0.01	4405	0.011	1.88	0.01	4210	0.004	14.85
	0.005	5350	0.011	1.54	0.005	5649	0.004	11.06
0 T	0.25	2656	0.009	4.65	0.25	2656	0.004	23.53

## 4 Discussion

The present study summarizes the results of Monte Carlo based Fano tests for two detector models in the presence of external magnetic fields. The charged particle transport in electro-magnetic fields is a challenge for every Class-II-Monte Carlo algorithm, because the particle trajectories are curved due to the Lorentz force, therefore the condensed history method in these codes may fail due to the choice of too large CH steps. Using the Fano test [15] under the conditions of external magnetic fields [14], [16] it can be checked, if the charged particle transport algorithm in a given geometry works properly. The Fano test is the only known method allowing the validation of charged particle energy deposition in heterogeneous media against an analytic expression, this way testing the charged particle step algorithm in the given geometry and also the bounding crossing algorithm.

The Fano tests in the present study were performed with the EGSnrc Monte Carlo code system [26] using the ‘simple’ EMF macros for the calculation of the charged particle trajectories in the presence of magnetic fields based on the work from Bielajew [12]. Within these macros the step size *s* can be influenced with the parameter EM ESTEPE and *s* is always proportional to the Larmor radius $r_{G}$ (see Eq. 6). That means, the step length is automatically adapted to varying particle energies and *B*-fields. But it is not clear, if one EM ESTEPE value can be used for all particle energies and magnetic field strength’s. The default value of EM ESTEPE is 0.020.

We performed Fano test for two detectors which are in widely clinical use, an air-filled ion chamber (PTW 31021) and a silicon diode (PTW 60016). In contrast to most other publications [4], [19], [20], [21] we did not only include the active volumes but all regions of the detectors in the Fano test. Moreover, we used very detailed models of both detectors made of up to fifty regions and did not simplify the detector models [11], [13]. The test was performed with different electron energies, covering a broad range of clinically used energies.

First of all, the results show, that not only the macros from Malkow and Rogers [13] within the EGSnrc code package are able to describe the charged particle transport in the presence of electro-magnetic fields adequately but also the older EMF macros from Bielajew [12]. By reducing the step size parameter EM ESTEPE in our simulations, we could reach a deviation of the Monte Carlo based and the analytical dose values in both detectors and all detector regions below 0.1%. For deviations less than this value the test is considered passed.

By comparing the results for the diode and the ion chamber, it was found that the diode can succeed the Fano test for much larger EM ESTEPE values compared to the ionization chamber (see Figure 5, Figure 7). Moreover, the test was generally more successful in regions with higher densities, so a larger EM ESTEPE parameter can be applied for all solid state detectors, strongly reducing calculation times. This is clear, as the CH step length always depends on the mass density, i.e. the larger the density the smaller the step length for a given parameter EM ESTEPE.

In agreement with the results of Lee *et al.* [11], the study has shown that the deviation from theoretical values of the calculated dose with the EGSnrc magnetic field macro (emf_macros.mortran) depends on the energy of the electrons. Although the step length *s* is automatically adjusted via the Larmor radius $r_{G}$ with particle energy, this change is not enough to pass the Fano test. For electron energies of 0.1 MeV the parameter EM ESTEPE had to be chosen as small as 0.005. Using high energy electrons as radiation source in the Fano cavity test leads to a good agreement with the theoretical dose values, even for a relative high EM ESTEPE value of 0.1. However, it should be noted that under real conditions of a bremsstrahlung’s photon field, the low energy electrons cause most of the dose in a patient or in a detector. The results of this study have highlighted the importance of the choice of the energy of the initial electrons in a Fano test in order for it to retain its validity in a realistic radiation field.

An important factor for all Monte Carlo codes is the calculation efficiency ∊. Restricting the step length of the CH steps will always reduce ∊, i.e. the CPU time for the calculation increases for the same type-A uncertainty. We did not perform a direct comparison of the efficiency of both electro-magnetic field macros (EMF and EEMF) which are available for the EGSnrc code. But, for the diode the efficiency was reduced only by about 25% comparing the simulations with and without a B-field (see Table 3) and an EM ESTEPE value of 0.025, resulting in deviations below 0.1% in all regions. Regarding the calculation efficiency in an ion chamber, Malkov and Rogers [13] state, that the calculation time increases by about 50% using their EEMF macros and a simplified model of a NE2571 chamber. Our results for the PTW 31021 chamber show an increase of the calculation time of about a factor of 2.5 if the EMF macro is applied and a EM ESTEPE value of 0.01 is chosen. This value was necessary to pass the Fano test for the ion chamber (see Fig. 6 and Table 3). That means, the newer EEMF macros from Malkov and Rogers seem to be much more efficient than the older EMF macros, but one has to keep in mind, that in the present study a much more detailed chamber model was used, and the calculation was performed until in every region the 0.1% level was reached. If it is indeed necessary to perform the Fano test not only for the active region but also for all adjacent regions is not quite clear. Looking at the results of the Fano test for all regions of a detector model, it can be seen that the deviations from the theoretical value are different in magnitude for different region. Consequently, one cannot conclude from the result of the Fano test of a single region to the remaining regions. However, the sensitive volume of the detector models of this study had the largest deviation from the expected value. When a decrease of the deviations in the sensitive volume could be achieved, this was accompanied by a decrease of the deviations in the other regions.

According to our study we recommend to use EM ESTEPE = 0.01 for the ionization chambers to pass the Fano test and for diodes the step size restriction EM ESTEPE can be chosen as 0.1. Additionally, it was found that the result of the Fano test depends on the primary electron energy of the Fano source. Therefore, it is recommended to choose an energy for the Fano radiation source according to the subsequent simulation task. This ensures that the Monte Carlo-based model has been evaluated for the radiation spectrum of interest. Otherwise, errors could occur that were not visible in the Fano cavity test.

## 5 Conclusion

With the increasing use of MR-linacs in modern radiotherapy, Monte Carlo based studies of the radiation transport in the presence of an external magnetic field are becoming increasingly important. Fano cavity tests especially for gas-filled detectors are highly recommended notably if these detectors are simulated in external magnetic fields. The present study has shown, that the older EMF macro, which is part of the EGSnrc code system is able to describe adequately the charged particle transport in external magnetic fields if the step size parameter EM ESTEPE is adequately chosen. For an external magnetic field $\overset{\to }{B}$ = 1.5 T, step size parameters EM ESTEPE = 0.1 for the diode and EM ESTEPE = 0.01 for the ion chamber yielded good Fano test results, i.e. deviations below 0.1% between Monte Carlo based and analytical dose values. As the Fano test results and the adequate step size depends on the primary electron energy, the Fano test should always be performed for several energies covering the whole range of clinical used energies.

## Declaration of Competing Interest

The authors declare that they have no known competing financial interests or personal relationships that could have appeared to influence the work reported in this paper.
